# Prevalence, Awareness, Treatment, and Control of High Blood Pressure: A Population-Based Survey in Thai Nguyen, Vietnam

**DOI:** 10.1371/journal.pone.0066792

**Published:** 2013-06-27

**Authors:** Duc Anh Ha, Robert J. Goldberg, Jeroan J. Allison, Thang Hong Chu, Hoa L. Nguyen

**Affiliations:** 1 Ministry of Health, Hanoi, Vietnam; 2 Department of Quantitative Health Sciences, University of Massachusetts Medical School, Worcester, Massachusetts, United States of America; 3 Thai Nguyen Department of Health, Thai Nguyen, Vietnam; 4 Institute of Population, Health and Development, Hanoi, Vietnam; 5 Oxford University Clinical Research Unit, Hochiminh City, Vietnam; College of Pharmacy, University of Florida, United States of America

## Abstract

**Background:**

Cardiovascular disease (CVD) is one of the leading causes of morbidity and mortality in Vietnam and hypertension (HTN) is an important and prevalent risk factor for CVD in the adult Vietnamese population. Despite an increasing prevalence of HTN in this country, information about the awareness, treatment, and control of HTN is limited. The objectives of this study were to describe the prevalence, awareness, treatment, and control of HTN, and factors associated with these endpoints, in residents of a mountainous province in Vietnam.

**Methods:**

Data from 2,368 adults (age≥25 years) participating in a population-based survey conducted in 2011 in Thai Nguyen province were analyzed. All eligible participants completed a structured questionnaire and were examined by community health workers using a standardized protocol.

**Results:**

The overall prevalence of HTN in this population was 23%. Older age, male sex, and being overweight were associated with a higher odds of having HTN, while higher educational level was associated with a lower odds of having HTN. Among those with HTN, only 34% were aware of their condition, 43% of those who were aware they had HTN received treatment and, of these, 39% had their HTN controlled.

**Conclusions:**

Nearly one in four adults in Thai Nguyen is hypertensive, but far fewer are aware of this condition and even fewer have their blood pressure adequately controlled. Public health strategies increasing awareness of HTN in the community, as well as improvements in the treatment and control of HTN, remain needed to reduce the prevalence of HTN and related morbidity and mortality.

## Introduction

Vietnam is in an epidemiological transition. The overall morbidity and mortality from non-communicable diseases (NCDs) in this country has been rising rapidly over the last two decades and the NCDs have become a major societal problem. Data from the national Ministry of Health (MOH) in 2010 showed that morbidity from NCDs was approximately 3 fold higher than that of infectious diseases [1].The changing epidemiologic profile of disease in Vietnam can be attributed to changes in the size and socio-demographic characteristics of the population as well as to increases in life expectancy. Increased life expectancy invariably prolongs the life-course exposure to cardiovascular disease (CVD) risk factors, rendering the population more susceptible to diseases of the heart and circulation. Indeed, CVD is the leading cause of death in Vietnam, accounting for approximately one quarter of all deaths annually and nearly one-fifth of the total burden of disease in Vietnam in 2008 [2]. A national survey in 2008 found that the prevalence of hypertension was 25% among persons 25 years and older [3]. The Vietnam National Health Survey (VNHS) in 2002 estimated that, by 65 years of age, nearly one half of all adult men and women will develop hypertension [4].

Despite its magnitude, hypertension is one of the most preventable risk factors for CVD; it can be easily detected and it can be effectively treated with low-cost drugs. Unfortunately, hypertension awareness, treatment, and control are unacceptably low in many countries, particularly in developing countries [5], [6]. Identifying factors associated with the awareness, management, and control of elevated BP is crucial to preventing the morbidity and mortality associated with hypertension. However, in Viet Nam, information on the socio-demographic and clinical factors associated with these endpoints is extremely limited and dated.

The objectives of the present observational study were to describe the prevalence, awareness, treatment, and control of hypertension, and to examine factors associated with these endpoints, among the adult population residing in Thai Nguyen province, a northern mountainous region of Vietnam.

## Methods

### Study Design

The present study used data collected in a population-based survey that was carried out among residents of Thai Nguyen, a province in the northern mountainous region of Viet Nam, in mid-2011 (population = 1,131,000, census 2009). A multistage stratified cluster sampling technique was utilized to ensure the representation of ethnic minorities living in remote areas and the poor. In the first stage of sampling, 60 communes were randomly selected from 180 communes throughout Thai Nguyen, using the probability proportionate to size selection procedure. The second stage consisted of selecting 2 villages randomly from each of the sampled communes. The third stage chose a random sample of 24 adult respondents (age≥18 years) from a list of adults living in the sampled village. This sampling strategy generated a sample size of 2,880 potential respondents. Since the prevalence of hypertension in adults less than 25 years old is low in Vietnam, we restricted the present analysis to adults 25 years and older.

### Data Collection

All eligible participants completed a structured questionnaire and dietary assessment. Information was collected about participants' age, sex, ethnicity (Kinh vs. minority groups), marital status, location of residence, education level, occupation, income (quintile groups were defined based on monthly income: (1)1–25 US$, (2)26–50, (3)52–75, (4)78–130, and (5)131–1,500), insurance status (yes vs. no), type of insurance (public vs. private), medical history, smoking, and eating habits. Community health workers performed a physical examination on all participants, which included the standardized measurement of weight, height, and blood pressure (BP). All local staff were trained in the standardized measurement of BP. Two consecutive readings of BP were taken on both arms with the participant in a seated relaxed position. The Omron HEM-790IT Automatic Blood Pressure Monitor with Advanced Omron Health Management Software was used to measure BP. The average of the two BP measures was used for purposes of analysis. Local health workers were carefully trained regarding the measurement of BP and the in-person interview survey using a structured questionnaire.

Hypertension was defined according to the Vietnamese Guidelines on Prevention and Control of Hypertension and the Sixth Joint National Committee on Prevention, Detection, Evaluation, and Treatment of High BP guidelines. Hypertension (HTN) was considered to be present: (1) if the systolic BP was ≥140 mmHg or diastolic BP was ≥90 mmHg for participants without diabetes and chronic kidney disease; (2) if the systolic BP was ≥130 mmHg or diastolic BP was ≥80 mmHg for participants with one of these two conditions; (3) if the participant reported a history of HTN; or (4) the participant reported taking anti-hypertensive medications (e.g., a diuretic, calcium channel blockers, beta-blockers, angiotensin-converting-enzyme inhibitors, or angiotensin II receptor blockers) during the prior 2 weeks Awareness of HTN was defined as participant's self-report of any previous diagnosis of HTN by a healthcare professional. The treatment of HTN was defined as self-reported use of a prescription medication (e.g., diuretics, angiotensin converting enzyme inhibitors) for the management of HTN during the previous 2 weeks. Control of HTN was defined as pharmacological treatment of HTN with a BP<140/90 mmHg for those without diabetes and chronic kidney disease, or a BP<130/80 mmHg for those with either one of these two conditions.

Participants who were aged <25 years at the time of the in-person interview, or who did not complete the questionnaire survey, were excluded from the present analysis. The primary outcomes of interest were the prevalence of HTN, awareness, treatment, and control of HTN.

### Data Analysis

We described our study population in terms of its socio-demographic, clinical, and lifestyle characteristics using simple descriptive statistics. Data were presented as percentages for categorical variables and median (inter quartile range- IQR) for continuous variables. The distribution of BP levels (median and IQR) in the study sample was examined according to select participant's characteristics, and compared using Wilcoxon-sum rank or Kruskal-Wallis tests.

The overall means (95% CIs) of systolic and diastolic blood pressure were calculated using survey (svy) procedures in STATA taking into account the multistage stratified cluster sampling technique that we used in the present study. The prevalence (95% CI) of HTN was described for the total study population and according to participant's socio-demographic, and behavioral characteristics using svy procedures. Logistic regression models (svy procedures) were utilized to examine factors associated with being hypertensive, and awareness of the condition. These factors were chosen based on the results of prior studies including age (25–39 years, 40–59, ≥60 years), sex, ethnicity (King vs. ethnic minority), educational level (primary, junior secondary, senior secondary and vocational/university), occupation (not working, agriculture work, and non-agriculture work), income (quintiles), location (urban vs. rural), BMI (normal, underweight, and overweight), smoking (ever vs. no), and must have salt when eating (yes vs. no), which have shown to be associated with our principal study outcomes. All analyses were performed using STATA 11.0 with (StataCorp. TX).

The study protocol was approved by the Population Council, New York, the Ethics Committee of the Institute of Population, Health, and Development (PHAD), Hanoi, and Thai Nguyen Department of Health, Vietnam. Written informed consent was obtained from all participants.

## Results

### Study Population Characteristics

The study sample consisted of 2,348 adult men and women residing in Thai Nguyen province, Vietnam, who were, on average, 45 years old. In this sample, 42% were men and 63% were of Kinh ethnicity. Overall, the majority of the study population was less than 60 years old, worked in the agricultural sector, lived in a rural area, had a normal body mass index (BMI), never smoked, and preferred having salt with meals (Table 1).

**Table 1:** Study Population Characteristics.

	n	%
Age (yrs)		
25–39	909	38.8
40–59	1,082	46.0
≥60	355	15.2
Sex		
Female	1,321	56.5
Male	1,018	43.5
Ethnicity		
Kinh	1,475	62.7
Ethnic minority	876	37.3
Education		
Primary	474	20.3
Junior Secondary	1,244	53.3
Senior Secondary	383	16.4
Vocational/university	231	10.0
Occupation		
Not working	399	17.0
Agricultural work	1,527	65.0
Non-agricultural work	425	18.0
Quintile of income		
1	464	22.0
2	537	25.6
3	278	13.2
4	403	19.1
5	423	20.1
Location		
Urban	403	17.2
Rural	1,943	82.8
Body mass index (BMI)*		
Normal (18.5-<25)	1,714	74.0
Underweight (<18.5)	469	20.3
Overweight (≥25)	131	5.7
Ever smoked cigarettes		
No	1,603	68.3
Yes	746	31.7
Must have salt when eating		
No	581	24.8
Yes	1,763	75.2

* BMI was calculated as weight(kg)/height(m)^2^.

Proportions were calculated using svy procedures.

### Distribution of Blood Pressure, Overall and according to Selected Participant's Characteristics

**Figure 1:**
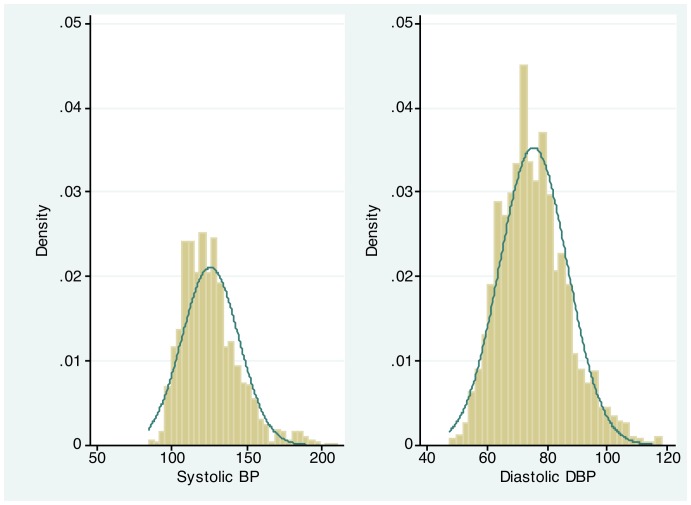
Overall Distribution of Participant's Blood Pressure.

**Figure 2:**
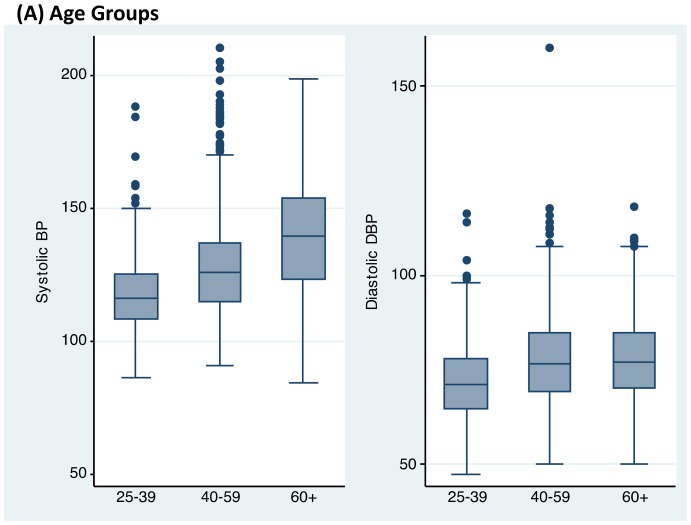
Distribution of Participant's Blood Pressure according to Selected Characteristics. Shown was distribution of systolic and diastolic of blood pressure according to selected participant's characteristics including age (Fig. 2A), sex (Fig. 2B), BMI categories (Fig. 2C), resident location (Fig. 2D), and educational level (Fig. 2E).

The overall mean of SBP was 125 mmHg (95% CI: 124–126 mmHg) and DBP was 75 mmHg (95% CI: 74–76 mmHg). In examining the distribution of SBP and DBP in our study sample, the median SBP was 123 mmHg (Inter-quartile range: 112–134 mmHg) and DBP was 75 mmHg (68–82 mmHg) (Figure 1). Both SBP and DBP increased significantly with advancing age and were significantly higher in men compared with women (p<0.001) (Figure 2 AB). Overweight participants were more likely to have higher levels of SBP and DBP compared with underweight and normal weight participants (Figure 2C). Participants with lower levels of education (primary) had higher levels of SBP compared to those with higher education levels (p<0.001), whereas DBP did not differ according to level of education (Figure 2E).

### Prevalence of Hypertension, Overall and according to Selected Participant's Characteristics

The overall prevalence of HTN in this population was 23.3% (95%CI: 21.1%–25.8%). The prevalence of HTN was higher in older individuals, men, Kinh, individuals with lower education, those who were not working, persons with higher income, those living in urban areas, and in those who had ever smoked or were overweight compared with respective comparison groups (Table 2)

**Table 2:** Prevalence, Awareness, Treatment, and Control of Hypertension (HTN) (95%CI) according to Selected Population Characteristics.

	HTN (n = 535)	Awareness of HTN (n = 173)	Treatment among persons who knew they had HTN (n = 72)	Control among persons received treatment (n = 27)
Age group (yrs)				
25–39	7% (5–11)	19% (8–39)	23% (5–61)	0% (0–0)
40–59	26% (22–30)	27% (21–35)	42% (30–56)	53% (33–72)
≥60	53% (47–58)	47% (38–56)	46% (31–62)	32% (17–51)
Sex				
Female	19% (16–22)	34% (25–43)	46% (28–66)	26% (12–47)
Male	30% (26–34)	34% (26–42)	41% (29–54)	51% (31–71)
Ethnic				
Kinh	24% (21–27)	33% (27–40)	48% (35–61)	38% (25–54)
Ethnic minority	22% (18–26)	38% (28–49)	28% (15–46)	42% (24–62)
Education				
Primary	36% (29–44)	30% (22–40)	49% (28–70)	59% (28–84)
Junior Secondary	18% (16–21)	36% (27–45)	42% (26–60)	36% (16–62)
Senior Secondary	25% (19–31)	35% (22–50)	31% (14–55)	40% (11–78)
Vocational/university	22% (16–31)	33% (20–50)	53% (37–68)	21% (6–53)
Occupation				
Not working	44% (38–50)	51% (41–61)	44% (31–59)	35% (20–54)
Agricultural work	18% (15–21)	24% (18–32)	40% (25–57)	31% (14–54)
Non-agricultural work	18% (14–24)	20% (13–31)	46% (20–74)	73% (32–94)
Quintile of income				
1	18% (14–23)	23% (12–39)	39% (21–61)	49% (14–85)
2	21% (16–27)	31% (19–46)	55% (31–76)	56% (26–83)
3	20% (15–28)	38% (20–60)	15% (4–42)	27% (5–73)
4	22% (16–29)	42% (31–53)	52% (32–71)	25% (9–55)
5	22% (18–27)	26% (15–41)	36% (15–65)	16% (3–56)
Location				
Urban	26%(22–31)	38%(28–50)	42%(27–63)	44%(23–64)
Rural	22%(19–25)	32%(25–38)	43%(30–56)	37%(24–52)
Body mass index (BMI)*				
Normal (18.5-<25)	22%(20–25)	33%(26–41)	44%(34–55)	36%(22–54)
Underweight (<18.5)	21%(17–25)	29%(19–41)	44%(22–69)	71%(34–92)
Overweight (>25)	46%(34–58)	45%(29–63)	41%(18–69)	20%(5–54)
Ever smoked				
No	20%(17–22)	31%(24–40)	52%(36–64)	32%(18–51)
Yes	32%(28–36)	37%(28–47)	33%(54–78)	50%(29–71)
Must have salt when eating				
No	23%(19–28)	47%(33–61)	27%(12–50)	22%(6–57)
Yes	23%(21–26)	30%(28–47)	50%(40–61)	43%(29–57)

* BMI was calculated as weight(kg)/height(m)^2^.

Proportions (95%CIs) were calculated using svy procedures.

### Awareness of Hypertension, Overall and according to Selected Participant's Characteristics

Among those with HTN, only 33.8% (95%CI: 28.1%–40.0%) were aware that they had elevated BP. Among participants diagnosed with HTN, older persons, minorities, and persons with higher education, those who were not working, who had a higher income, were living in urban areas, had a higher BMI, or those who had ever smoked were more likely to be aware of their HTN than respective comparison groups (Table 2). Persons who reported “must have salt when eating” were more likely to know that they had HTN compared with those who did not report knowledge of their condition.

### Prevalence of Hypertension Treatment, Overall and according to Participant's Characteristics

Only 43.2% (95%CI: 32.6%–54.5%) of participants who were aware of their HTN received treatment for this condition as measured by taking any anti-hypertensive medication during the last 2 weeks before the survey. Among participants who knew they had HTN, the prevalence of treatment was higher in older persons, women, and Kinh ethnicity compared with younger persons, men, and minorities (Table 2). Persons with vocational or university education, worked in other areas than agriculture, at the 2^nd^ quintile income, were living in town, who had a normal BMI, never smoked, and reported “must have salt when eating” were more likely to take prescribed hypertensive medications compared with respective comparison groups.

### Prevalence of Controlled Hypertension, Overall and according to Participant's Characteristics

Among participants who were treated with anti-hypertensive medications, only 38.8% (95%CI: 26.8%–52.3%) had their BP controlled. Among participants receiving HTN treatment during the last 2 weeks, persons between 40–59 years old, men, persons with lower educational attainment (primary), those working in the non- agricultural sector, at the 2^nd^ quintile income, who were living in urban areas, and those who were classified as being underweight, who smoked cigarettes, and reported “must have salt when eating” were more likely to have their BP controlled compared with respective comparison groups (Table 2).

### Factors Associated with Prevalence, Awareness, Treatment and Control of Hypertension

Results from our multivariable regression analysis showed that older individuals (≥40 years), men, and being overweight were significantly associated with a greater likelihood of being diagnosed with HTN. On the other hand, higher educational level (≥junior secondary school) and being underweight were associated with a lower risk of HTN (Table 3). Persons who worked both in agricultural and non-agricultural sectors were less likely to be aware of their HTN whereas persons who were classified as being overweight and had ever smoked were more likely to know that they had HTN compared to those who did not have these characteristics (Table 3). Since the number of persons who received anti-hypertensive medication and had their BP controlled was small, factors associated with these conditions were not examined in regression analyses.

**Table 3:** Factors Associated with Prevalence and Awareness of Hypertension.

	Hypertension (n = 535)		Awareness (n = 173)	
	Adjusted† OR (95%CI)	p-values	Adjusted† OR (95%CI)	p-values
Age group (yrs)				
25–39	1.00		1.00	
40–59	**4.92(2.84–8.50)**	**<0.001**	1.59(0.33–7.74)	0.56
≥60	**12.27(6.04–24.91)**	**<0.001**	4.62(0.77–27.87)	0.09
Sex				
Female	1.00		1.00	
Male	**1.85(1.12–3.07)**	**0.017**	0.37(0.12–1.13)	0.81
Ethnic				
Kinh	1.00		1.00	
Ethnic minority	1.17(0.82–1.68)	0.37	1.62(0.92–2.90)	0.10
Education				
Primary	1.00		1.00	
Junior Secondary	**0.41(0.25–0.68)**	**0.001**	1.80(0.83–3.86)	0.13
Senior Secondary	**0.58(0.34–0.97)**	**0.038**	3.03(0.85–10.78)	0.09
Vocational/university	**0.44(0.20–0.96)**	**0.041**	1.62(0.58–4.56)	0.36
Occupation				
Not working	1.00		1.00	
Agricultural work	0.62(0.27–1.43)	0.26	**0.31(0.12–0.79)**	**0.016**
Non-agricultural work	0.89(0.56–1.42)	0.62	**0.35(0.16–0.77)**	**0.010**
Quintile of income				
1	1.0		1.0	
2	1.57(0.85–2.91)	0.14	2.27(0.85–6.18)	0.11
3	1.32(0.69–2.54)	0.40	1.28(0.33–4.94)	0.71
4	1.49(0.67–3.34)	0.32	1.38(0.33–5.55)	0.65
5	1.29(0.73–2.29)	0.37	0.63(0.15–2.73)	0.52
Location				
Urban	1.0		1.0	
Rural	1.32(0.82–2.10)	0.24	1.36(0.63–2.93)	0.44
Body mass index (BMI)*				
Normal (18.5-<25)	1.00		1.00	
Underweight (<18.5)	**0.66(0.45–0.98)**	**0.040**	0.80(0.33–1.95)	0.62
Overweight (>25)	**3.47(1.89–6.35)**	**<0.001**	**2.79(1.11–6.98)**	**0.028**
Ever smoked cigarettes				
No	1.00		1.00	
Yes	1.25(0.78–2.00)	0.35	**3.04(1.02–9.04)**	**0.046**
Must have salt when eating				
No	1.00		1.00	
Yes	1.16(0.76–1.77)	0.49	0.55(0.24–1.26)	0.15

* BMI was calculated as weight(kg)/height(m)^2^.

† Multivariable logistic regression models with svy procedures in STATA.

## Discussion

The results of our study suggest that nearly one in every four adults in Thai Nguyen are hypertensive. The prevalence of HTN in residents of this province was slightly lower than that of the whole country of Vietnam in a national survey carried out in 8 Vietnamese provinces and cities, but slightly higher than the prevalence of HTN observed among those from the highland region in the same study [3]. The prevalence of awareness and treatment of HTN were also lower, while the control of hypertension was slightly higher, compared with the national average [3]. Despite these relatively small between study differences, the collective results of these population-based surveys demonstrate that the level of awareness, treatment, and control of HTN in Thai Nguyen remains disproportionately and unacceptably low. These findings are similar to the results observed in many developing countries including China, Thailand and India [7], [8], [9], [10], [11], [12].

### Prevalence of Hypertension, Awareness, Treatment, and Control

The findings of our study indicate that hypertension awareness and management was far from optimal, a disturbing trend that has been observed in many other developing countries [6], [10], [11], [13]. For example, a recent study from Guangdong southern China showed that one fifth of this population had HTN and the awareness, treatment, and control of HTN in hypertensive persons living in rural regions were 17.6%, 10.4%, and 3.4%, respectively, which were lower than the corresponding figures in urban regions (42,8%, 37.9%, and 13.5%, respectively) [10]. Similar data were reported from the most recent Thai national survey in 2009 [11]. In the 1970's, based on the findings from several BP surveys in the United States, researchers developed the “rule of halves” stating that half the hypertensive population is undetected, half of those detected are untreated, and in half of those treated hypertension is not controlled [14], [15]. Currently, the “rule of halves” is no longer applicable in developed countries, and has been replaced by the “rules of two-thirds” as a result of increased awareness and control of HTN [16], [17], [18]. However, our results showed that the awareness, treatment, and control of HTN in Thai Nguyen are far from approaching the “rule of halves”. Indeed, the detection rate of HTN in our population is very low. It will continue to be challenging to reach the ’rule of halves” in this population unless comprehensive strategies are going to put into practice for the management of HTN in Thai Nguyen in the near future. These measures would include both broad policy measures supplemented by Thai Nguyen Department of Health and the Ministry of Health, Vietnam.

### High Risk Groups for Hypertension

Our study demonstrated that the prevalence of HTN was higher in older persons, men, individuals with lower education (primary), with higher income, living in urban areas or town, those who were overweight, and who had ever smoked; these findings are consistent with the results from previous studies [7], [12], [19], [20]. After adjustments for other variables, we found that older age, male sex, lower education level, and overweight were independently associated with an increased risk of HTN, confirming that our population has shared the same established risk factors found in other countries [7], [18], [19], [20], [21], [22]. For example, data from a recent national survey in Saudi Arabi found that significant predictors of HTN included male sex, urbanization, low education, low physical activity, obesity, diabetes, and hypercholesterolemia [20]. Although the prevalence of overweight is still low in the Vietnamese population, being overweight was associated with an increased risk of being diagnosed with HTN. As the Vietnamese population is aging, and the prevalence of overweight increases, likely due to an increasing adoption of western diets and lifestyle practices, it is expected that the prevalence of HTN will continue to rise if no effective, community-based interventions will be implemented in the future.

Our findings also indicated that overweight persons are more likely to be aware that they are hypertensive compared with those having normal body weight; these findings are consistent with the results from other recent studies conducted in both developing and developed countries [3], [5], [23], [24], [25]. In addition to the well-known adverse health effects of overweight and obesity on the development of HTN, evidence has shown that in developing countries, like Vietnam, body weight is positively associated with socio-economic status (SES) [26], and individuals with higher SES are more likely to access health care services compared to those of lower SES. In addition, data from our present study suggested that persons who have ever smoked cigarettes were more likely to be aware of their condition than those who had never smoked. This finding was similar to results of the Jordan National Survey in 2009, which found that awareness of HTN was positively associated with older age, smoking, and diabetes [19]. Furthermore, our study found that persons who worked were less likely to be aware that they were hypertensive compared with those who did not work. This consistent with findings from the study of multi-ethnic Asian population between 2004 and 2007 in Singapore, which indicated that reduced awareness and treatment of HTN were associated with being younger, never married and working adults with a higher education level [27].

### Public Health Implications

Our findings contribute to the ongoing policy debate with respect to the prevention and control of HTN in Vietnam, especially for the current National Targeted Program for Prevention and Control of Cardiovascular disease. Results from the present study, similar to what has been found in a recent national study [3], showed that Vietnam in general, and Thai Nguyen in particular, are facing a high prevalence of HTN attributed morbidity and mortality. However, community-based interventions for enhancing the awareness and control of HTN remain very limited in Vietnam, though with potentially encouraging results. Indeed, a recent study conducted in a rural province of northern Vietnam demonstrated that a community-based model for comprehensive HTN care using both top-down and bottom-up approaches was found to be both feasible and acceptable in this setting [28]. More community-based intervention approaches for HTN control should be investigated. In many countries, public health measures such as mass education campaigns, dietary and lifestyle interventions, and pharmacotherapy have been shown to be effective for alleviating the burden of HTN and improving its control [21], [29], [30], [31].

In order to better control HTN in Thai Nguyen and throughout the country, primary interventions including early detection and prompt treatment of HTN should be the central focus. Since all drugs used for treating hypertension and hyperlipidemia are now off-patent and available widely across the country, these programs should be relatively easily scaled up through primary health care or outpatient-clinics. Moreover, education programs including mass media approaches to reduce sodium intake, which has been highly recommended by the WHO [32], and proven to be cost-effective in Vietnam [33], as well as newer patient education approaches, such as culturally appropriate storytelling to control blood pressure [34] need to be implemented throughout the country.

### Study Strengths and Limitations

The strengths of the present study includes the population-based multistage stratified cluster sampling design which enhances the generalizabilty of the present findings as well as the careful measurement of BP and its possible predisposing factors. However, the study has several limitations that must be kept in mind in interpreting the study results. First, although study staff were well trained in the measurement of BP, we may have overestimated the prevalence, and underestimated the control of elevated BP, since BP levels presented were based on the average of two measurements performed by a local health worker at a single clinic visit [35]. Second, information on a history of HTN and use of anti- hypertensive medication was based on self-reported data; inasmuch, misclassification of the prevalence, awareness, treatment, and control of HTN may have occurred. Lastly, due to small numbers of participants who received treatment and had their BP controlled, we were unable to identify factors associated with these endpoints.

## Conclusions

In conclusion, the results of this population-based study indicate that HTN is an important public health problem in Thai Nguyen. Comprehensive intervention strategies that target the general population, and focus on raising the awareness and treatment of the condition and its risk factors, must be put in place in Thai Nguyen to reduce the burden of HTN related disease. Regular surveys in different provinces throughout Vietnam are needed to continually monitor trends in HTN prevalence and factors associated with HTN awareness and management to better inform future interventions aimed at reducing the magnitude and improving treatment and control of this serious condition.
