# Correction: Prevalence, Awareness, Treatment, and Control of High Blood Pressure: A Population-Based Survey in Thai Nguyen, Vietnam

**DOI:** 10.1371/annotation/bbf4cb48-2f22-49ba-afce-b5263799c592

**Published:** 2014-01-02

**Authors:** Duc Anh Ha, Robert J. Goldberg, Jeroan J. Allison, Thang Hong Chu, Hoa L. Nguyen

Figures 2B through 2E do not appear.

Please view Figure 2B here: http://www.plosone.org/corrections/pone.0066792.g002b.cn.tif

Please view Figure 2C here: http://www.plosone.org/corrections/pone.0066792.g002c.cn.tif

Please view Figure 2D here: http://www.plosone.org/corrections/pone.0066792.g002d.cn.tif

Please view Figure 2E here: http://www.plosone.org/corrections/pone.0066792.g002e.cn.tif

